# Integration of reconfigurable microchannels into aligned three-dimensional neural networks for spatially controllable neuromodulation

**DOI:** 10.1126/sciadv.adf0925

**Published:** 2023-03-10

**Authors:** Sohyeon Jeong, Hyun Wook Kang, So Hyun Kim, Gyu-Sang Hong, Min-Ho Nam, Jihye Seong, Eui-Sung Yoon, Il-Joo Cho, Seok Chung, Seokyoung Bang, Hong Nam Kim, Nakwon Choi

**Affiliations:** ^1^ Brain Science Institute, Korea Institute of Science and Technology (KIST), Seoul 02792, Korea.; ^2^ Division of Bio-Medical Science and Technology, KIST School, University of Science and Technology (UST), Seoul 02792, Korea.; ^3^ MEPSGEN Co. Ltd., Seoul 05836, Korea.; ^4^ School of Mechanical Engineering, Korea University, Seoul 02841, Korea.; ^5^ SK Biopharmaceuticals Co. Ltd., Seongnam 13494, Korea.; ^6^ Department of Life Sciences, Korea University, Seoul 02841, Korea.; ^7^ KHU-KIST Department of Converging Science and Technology, Kyung Hee University, Seoul 02453, Korea.; ^8^ Division of Nano and Information Technology, KIST School, University of Science and Technology (UST), Seoul 02792, Korea.; ^9^ Department of Biomedical Sciences, College of Medicine, Korea University, Seoul 02841, Korea.; ^10^ Department of Anatomy, College of Medicine, Korea University, Seoul 02841, Korea.; ^11^ KU-KIST Graduate School of Converging Science and Technology, Korea University, Seoul 02841, Korea.; ^12^ Department of Medical Biotechnology, Dongguk University, Goyang 10326, Korea.; ^13^ School of Mechanical Engineering, Yonsei University, Seoul 03722, Korea.; ^14^ Yonsei-KIST Convergence Research Institute, Yonsei University, Seoul 03722, Korea.

## Abstract

Anisotropically organized neural networks are indispensable routes for functional connectivity in the brain, which remains largely unknown. While prevailing animal models require additional preparation and stimulation-applying devices and have exhibited limited capabilities regarding localized stimulation, no in vitro platform exists that permits spatiotemporal control of chemo-stimulation in anisotropic three-dimensional (3D) neural networks. We present the integration of microchannels seamlessly into a fibril-aligned 3D scaffold by adapting a single fabrication principle. We investigated the underlying physics of elastic microchannels’ ridges and interfacial sol-gel transition of collagen under compression to determine a critical window of geometry and strain. We demonstrated the spatiotemporally resolved neuromodulation in an aligned 3D neural network by local deliveries of KCl and Ca^2+^ signal inhibitors, such as tetrodotoxin, nifedipine, and mibefradil, and also visualized Ca^2+^ signal propagation with a speed of ~3.7 **μ**m/s. We anticipate that our technology will pave the way to elucidate functional connectivity and neurological diseases associated with transsynaptic propagation.

## Teaser

Microfluidic channels integrated with aligned 3D neural networks enable spatiotemporally resolved neuromodulation.

## INTRODUCTION

Sophisticated yet anisotropically organized neural networks are indispensable routes to transmit signals within and between regions in the brain, including long-range communications regulated by neural connections (*1*–*3*). While the region-to-region connectivity remains largely unknown, the need to unravel such connectivity and to elaborate on specific brain functions such as memory (*4*, *5*) in more detail has persistently expanded in physiological and pathological contexts. In this regard, several emerging techniques have enabled region-specific brain stimulation and recording, predominantly through in vivo studies using mice and rats. For example, optogenetic, magnetomechanical, photothermal, ultrasonic, and nanopipette modulations have shown the capability to stimulate specific brain regions in animal models (*6*–*10*). Despite the promising feature, these techniques require additional preparation before experiments, such as genetic modifications, stimulation-applying devices, and nanoparticles. Alternatively, chemical stimulation is a simple but controllable approach to avoid leaving residues in tissue (*11*).

Although many studies for neuronal modulation have used in vitro culture models, most in vitro neural networks still rely on the two-dimensional (2D) culture on plastic dishes or coverslips. However, typical plastic dishes and coverslips provide neither mechanical properties nor topological cues relevant to the directional alignment of neural networks in vivo (*12*–*14*). The advent of nano- and micropatterning techniques initiated substantial fabrication of aligned neural networks but offered cellular microenvironments limited to 2D morphologies (*15*, *16*). Introducing the microphysiological system (MPS) revolutionized tissue mimetics regarding the in vivo–like mechanical properties, biomimicry of microenvironment, and coculture of multiple cell types (*17*–*20*). Sophisticated capabilities to engineer tissue microenvironment in the MPS can be tailored to specific tissues or organs, including the recapitulation of neural networks in 3D scaffolds (*21*, *22*).

Regarding the reconstruction of neural networks in vitro, neurons’ aligned morphology is critical because of their structural and functional characteristics in vivo: anisotropic organization and efficient transmittance of signals along neurites, respectively (*23*, *24*). Accordingly, applying a magnetic field (*25*), mechanical stretches (*26*), and the shear flow of a gel (*27*) allowed for guiding neurite growth. Previously, we also developed a guiding technique by manipulating an extracellular matrix (ECM) hydrogel during its sol-gel transition (*28*, *29*). However, these aligned neural networks are unsuitable for investigating neuromodulation due to the inability of localized delivery, transport (i.e., diffusion and convection) control, and full-thickness observation. More specifically, in conventional 2D or open-type 3D tissue models sharing a reservoir of culture medium, molecular diffusion rapidly leads to consequential dilution homogeneously throughout the medium, and, thus, the localized delivery (stimulation) and maintenance of “hotspots” have been unavailable (*30*, *31*).

In contrast, the molecular transport in microfluidic devices attributes spatiotemporal control of engineered tissue microenvironment to convection through microchannels and subsequent diffusion to surrounding cell-seeded scaffolds (*32*–*34*). Therefore, biochemical stimuli can be spatially localized and temporally evolving, and their persistent gradients sustain for a long time. Nevertheless, no technology platform has permitted spatiotemporal control of chemo-stimulation in anisotropic 3D neural networks in vitro.

Here, we present the seamless integration of perfusable microchannels into collagen fibril–aligned 3D neural networks by adapting a single fabrication principle with the reconfigurable deformation of an elastic device. Two key features of our technology platform are as follows:

1) Precompression of an elastomeric microfluidic chip temporarily closes the microchannels, preventing the permeation of sol-phase collagen. After the release of the precompression at a critical time point, partially gelated collagen sustains and reopened (“reconfigurable”) microchannels remain unfilled.

2) The release of the precompressed chip to its initial configuration during the sol-gel transition of collagen induces the collective rotation of microfibrils, ultimately aligning collagen fibrils in parallel to the compression axis.

Because these two key features identically share the deformation-induced fabrication principle, we could establish a robust and seamless platform for spatiotemporally resolved local neuromodulation in anisotropically organized 3D neural networks. We also verified the design and fabrication principles with computational simulations and experiments. Accordingly, we could demonstrate the local stimulation of potassium chloride (KCl) followed by propagation of neuronal Ca^2+^ signal along an anisotropically organized neural network in a monolithic 3D tissue. Furthermore, we also demonstrated spatiotemporally resolved neuromodulation with Ca^2+^ signal inhibitors such as tetrodotoxin (TTX), nifedipine, and mibefradil. We envision that our platform can pave the way to elaborate the identification of the functional connectivity between brain regions by local stimulation and signal observation.

## RESULTS

### Simultaneous and seamless integration of reconfigurable microchannels and collagen microfibril alignment

**Fig. 1.**
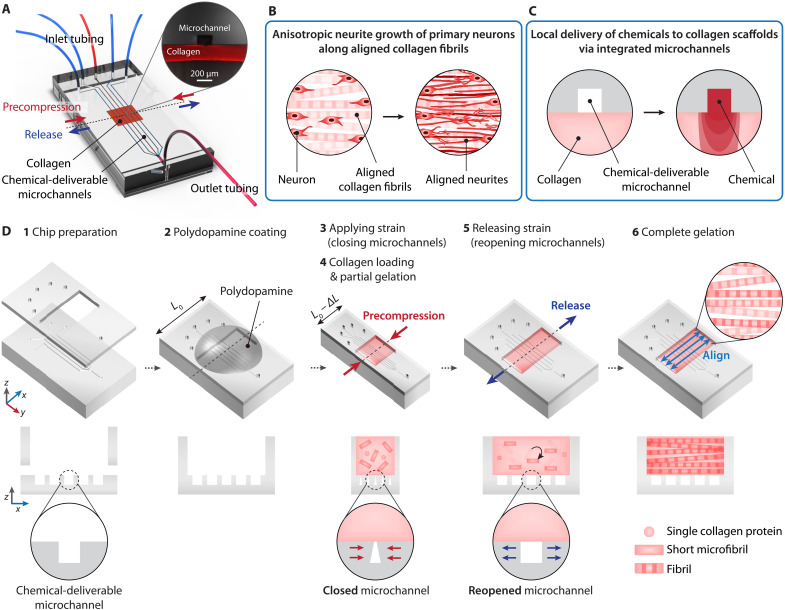
Overview of simultaneous integration of reconfigurable microchannels and alignment of collagen fibrils for spatially controllable chemical modulation in 3D neural networks in vitro. (**A**) A schematic illustration of the microchannel-integrated, anisotropically organized collagen 3D culture platform. An inset image shows a cross-sectional view along a dashed line. (**B** and **C**) Schematic illustrations displaying two key features: anisotropic neurite growth along aligned collagen fibrils (B) and local microfluidic delivery of chemicals (C). (**D**) Schematic illustrations showing fabrication process: preparation of a PDMS chip consisting of a 400-μm-thick top and a 10-mm-thick bottom layer patterned with microchannels (1), coating of polydopamine on the PDMS chip with the width of *L*_0_ (2), precompression to the width of *L*_0_ − ∆*L*, also leading to closing the microchannels (3), loading of sol-phase collagen followed by partial gelation (4), strain release, resulting in collective rotation of collagen microfibrils and reopening the microchannels (5), and complete gelation to achieve alignment of collagen fibrils in parallel to the precompression direction (i.e., the *x* axis) with seamless integration of the microchannels (6). The bottom panels depict cross-sectional views on the *xz* plane.

We first developed a fabrication technology to integrate reconfigurable microchannels with the alignment of collagen microfibrils simultaneously and seamlessly. This technology enabled us to manufacture an anisotropically organized 3D culture platform capable of spatiotemporal control of biochemical microenvironment (Fig. 1A). Our device exhibits two key features: (i) aligned collagen fibrils as 3D contact guidance for anisotropic neurite growth and (ii) microchannels for local (e.g., region-specific) delivery (i.e., convection-diffusion) of biochemicals to the directly contacting aligned neuronal networks (Fig. 1B).

We adopted the deformation-induced alignment technique of collagen type I fibrils in constructing an aligned neural network (*29*). Briefly describing, we precompress an elastomeric [polydimethylsiloxane (PDMS)] substrate with a width of *L*_0_ by ∆*L* to [*L*_0_ − ∆*L*] and load sol-phase collagen into a well of the substrate. After 5 min as a crucial period of partial fibrillogenesis (i.e., strain duration, *t*_d_), the release of the precompressed PDMS induces the collective rotation of short collagen microfibrils within the gel, aligning in parallel to the precompression axis. Then, the short microfibrils grow along the aligned orientation, forming an aligned 3D collagen matrix.

To integrate underlying microfluidic channels, we revamped the “capillary bursting valve” concept, regulating liquid flow by a variation of microchannel geometry (*35*), and invoked this strategy spontaneously applied during the collagen alignment steps stated above. More specifically, we started with preparing a partially open PDMS microfluidic chip by bonding a top layer (400 μm thick) with a rectangular through-hole, five inlet ports, and an outlet port onto a bottom layer (10 mm thick) patterned with five microchannels merging to one (e.g., initial width, *w*_0_, of 100 μm and depth, *d*, of 100 μm) (Fig. 1D1). After coating polydopamine on the inner surfaces of the open chamber (Fig. 1D2) to prevent undesirable delamination of collagen, we applied strain to the PDMS microfluidic chip along a direction (i.e., the *x* axis) perpendicular to a longitudinal axis of the microchannels (i.e., the *y* axis) (Fig. 1D3). Then, we loaded collagen and allowed for partial gelation (Fig. 1D4). During this precompression, all the microchannels with a rectangular cross section deformed into a triangular or trapezoidal shape because the open inter-ridge regions were more deformable than the much thicker base (i.e., most of the bottom layer). Accordingly, almost closed inter-ridge openings prohibited the penetration of sol-phase collagen, which corresponds to applying the capillary bursting valve principle. Then, the release of the compressive stress played a dual role in (i) reopening the microchannel ridges (recovering the microchannels’ original geometry) and (ii) aligning short collagen microfibrils (Fig. 1D5). We refer to the “reopening after closing” as “reconfigurable.” Over the following complete gelation for 30 min, collagen fibrils aligned across the entire construct (400 μm thick) without permeation into the microchannels (Fig. 1D6). Therefore, perfusable microchannels formed seamlessly underneath an aligned collagen matrix without additional steps.

### Underlying physics and design principles of reconfigurable microchannels in the maintenance of unfilled states during the patterning of collagen matrices

**Fig. 2.**
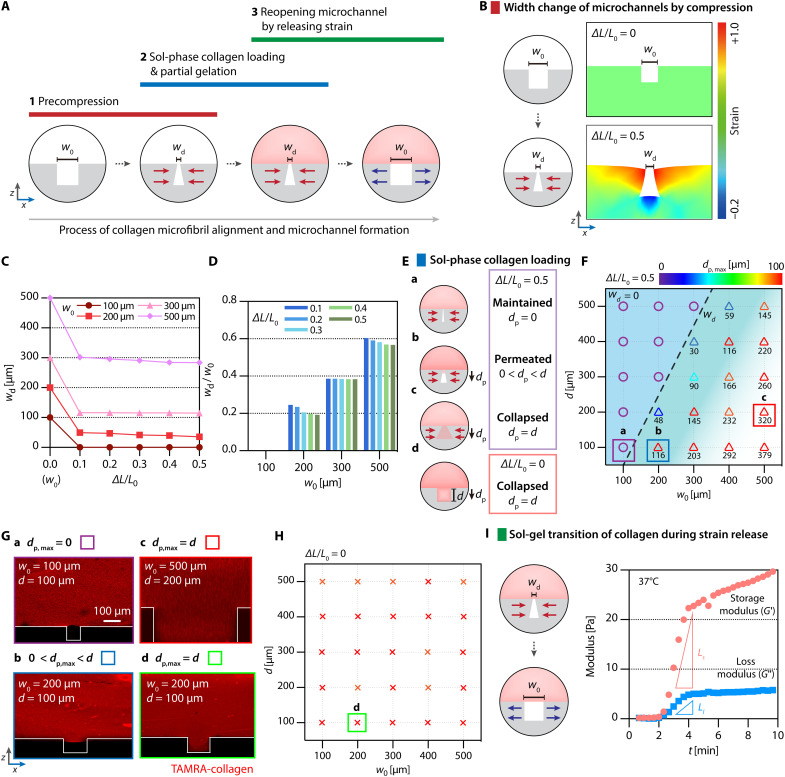
Design principles for integrating reconfigurable microchannels into collagen fibril–aligned 3D matrix. (**A**) Schematic snapshots representing three critical steps: (1) precompression (red), (2) sol-phase collagen loading and partial gelation (blue), and (3) reopening microchannel by releasing strain (green), where underlying physics and design principles should be considered. (**B**) A schematic illustration and computational calculations of 2D strain displaying nonisometric changes in cross-sectional width of a PDMS microchannel for the critical step (A1). (**C**) A plot of changes in the microchannel width depending on strain magnitude. Violet, pink, red, and dark red denote *w*_0_ of 100, 200, 300, and 500 μm, respectively. (**D**) A grouped bar graph of *w*_d_/*w*_0_ depending on *w*_0_. Blue, light blue, cyan, green, and dark green denote ∆*L*/*L*_0_ of 0.1, 0.2, 0.3, 0.4, and 0.5, respectively. (**E**) Schematic illustrations of loading sol-phase collagen onto precompressed (a to c) and uncompressed (d) microchannels, leading to three consequential states: maintained (*d*_p_ = 0), permeated (0 < *d*_p_ < *d*), and collapsed (*d*_p_ = *d*). *d*_p_ refers to the depth of gel permeation. (**F**) A zone-marked contour plot displaying computational predictions for permeation of sol-phase collagen at 5 min into deformed vacant microchannels under precompression with varying *w*_0_ and *d*: *d*_p_ = *w*_d_ = 0 (◯) and 0 < *d*_p_ < *d* (△). (**G**) Fluorescence images showing cross-sectional views of microchannels and collagen (red) under the boxed conditions indicated in (F) and (**H**). (H) A zone-marked contour plot displaying computational simulations for ∆*L*/*L*_0_ = 0. TAMRA, 5-carboxytetramethylrhodamine. (**I**) A schematic illustration for the critical step (A3) and a plot of storage (*G*′; red) and loss (*G*″; blue) moduli over an early period of fibrillogenesis of collagen (2.5 mg/ml) at 37°C. *L*_s_ and *L*_l_ denote slopes of the modulus curves.

For the localized biochemical modulation, microchannels should remain unfilled during the precompression and release of the PDMS microfluidic chip (Fig. 2A). To determine design criteria to prevent collagen permeation into a microchannel while aligning collagen fibrils simultaneously and seamlessly, we identified three critical processes along the fabrication streamline. More specifically, we should consider underlying physics during (i) the width change of microchannels by precompression (Fig. 2, B to D), (ii) the loading of sol-phase collagen onto the width-deformed microchannels (Fig. 2, E to H), and (iii) the reopening of microchannels underneath partially gelated collagen by strain release (Fig. 2I).

To do so, we performed computational simulations with fabrication parameters (i.e., *w*_0_ and *d*). First, we monitored the deformation extent of microchannel width (i.e., deformed width, *w*_d_) while varying both strain magnitude of the precompression [0 ≦ (∆*L*/*L*_0_) ≦ 0.5] and initial width (*w*_0_) (Fig. 2, B to D). As we apply the compressive strain directly on the PDMS chip, stress transmits to the microchannels’ open ends along the precompression axis and predominantly leads to secondary deformation of the open ends due to the free of physical constraint (Fig. 2B). Notably, the deformed width showed a marked reduction at a strain of 0.1, the lowest variation of the strain magnitude (Fig. 2, C and D). It remained almost constant for higher magnitudes regardless of the initial width. In addition, while the width ratio between open ridges before and after the deformation gradually decreased as the initial width decreased, *w*_0_ of 100 μm was utterly closed (i.e., *w*_d_ = 0) (Fig. 2D).

Because we loaded sol-phase collagen into the precompressed rectangular well, the strain applied to the partially gelated collagen during the release step would be identical to that applied to the rectangular well. Our computational simulation and experimental measurements indicated that the rectangular well with the precompressive axial length (*L*_0,well_) of 5 mm became 3.5 mm, revealing that the strain of collagen during the release step (|∆L|/*L*_0,well_) was 0.3. Because of the polydopamine coating on the inner surfaces of the well, we have not observed the delamination of the partially gelated collagen after the release.

Next, we investigated whether sol-phase collagen would permeate spontaneously into the open side of microchannels under the compression-induced deformation with ∆*L*/*L*_0_ of 0.5, depending on various combinations of *w*_0_ and *d*. As design criteria, we defined three consequent states of a microchannel with the depth of gel permeation, *d*_p_ (Fig. 2E): (a) maintained microchannel when *d*_p_ = 0, (b) partially permeated microchannel when 0 < *d*_p_ < *d*, and (c) collapsed microchannel when *d*_p_ = *d*. We simulated the gel permeation at 5 min after the loading of sol-phase collagen that had dynamic viscosity of 7.9 Pa·s (*36*, *37*) and mapped the wettability of microchannels’ inner surface by displaying computational predictions in a zone-marked graph (Fig. 2F). Sol-phase collagen showed no permeation in the microchannels (i.e., *d*_p_ = 0) when *w*_d_ = 0, which leads to an ideal regime for the maintained microchannels from nine combinations of *w*_0_ and *d* (purple circles in Fig. 2, F and Ga). Meanwhile, other combinations of the dimensions resulted in *w*_d_ > 0 and, thus, *d*_p_ > 0 to some extent up to *d*_p,max_ of 100 μm from our simulations (triangles in Fig. 2F and fig. S1). These cases fall into an empirical regime where we experimentally observed the partially permeated microchannel (Fig. 2Gb) or complete permeation causing the collapsed microchannel (Fig. 2Gc). Notably, we confirmed the functionality of microchannels with [*w*_0_ by *d*] of [100 μm by 100 μm] or [200 μm by 200 μm] for the following experiments in this study. In addition, these computational predictions and experimental results indicate that *w*_0_ is a more critical parameter than *d*, affecting empirical success (e.g., reproducibility) toward reducing *w*_d_ and, therefore, *d*_p_. From these computational predictions and our experimental data, an interfacial zone between the ideal and empirical regimes, where *w*_d_ is equal to or less than 116, would lead to the successful formation of functional microchannels. We emphasize that no precompression immediately led to collapsed microchannels (Fig. 2, Ed and H).

Our technique to integrate the formation of microchannels with the alignment of collagen fibrils exploits fibrillogenesis kinetics that essentially includes a sol-gel transition during partial gelation. Empirically, we found that this transition ranged from 5 to 15 min, which played a critical role in determining an optimized strain-releasing time, also referred to as strain duration, for the desired alignment of collagen microfibrils (*29*, *38*). To estimate the time scale of the sol-gel transition more analytically, we measured rheological properties (i.e., storage and loss moduli) of collagen type I (2.5 mg/ml) during fibrillogenesis at 37°C (Fig. 2I). The rheometer applied shear stress to a viscoelastic material (i.e., sol-phase collagen) and allowed for measurements of loss and storage moduli matching the applied stress. The storage modulus represents stored deformation energy, and the loss modulus represents the dissipated deformation energy when flowing.

While both storage (*G′*) and loss (*G*″) moduli overlapped almost identically until 2 min, confirming that collagen consisted dominantly of single proteins in a sol state. As the transition started, *G′* became higher than *G*″, which means that the collagen began to display a gel-like behavior. Accordingly, collagen scaffolds retain their shape because the elastic characteristic is superior to the viscous characteristic. Then, *G′* increased much more rapidly than *G*″, indicating the rapid nucleation of collagen proteins for generating short microfibrils. At ~4 min, changes in both *G′* and *G*″ over time (i.e., slopes of modulus curves: *L*_s_ and *L*_l_) reduced notably, implying the end of partial gelation and sequential transition to the fibril growth stage. In other words, during the fibrillogenesis of collagen type I, higher increments of the storage modulus compared to the loss modulus imply a bias of the viscoelastic collagen gel toward elastic properties. These rheological measurements were consistent with our previous experimental observations with the identical composition of collagen (*29*) and a study by others that reported similar trends over an extended time scale during fibrillogenesis of collagen (1 mg/ml) at 25°C (*39*). Overall, our consideration of underlying physics (i.e., gelation kinetics of collagen and mechanical deformation of microfluidic channels) sequentially orchestrated and enabled the fabrication of aligned collagen matrices integrated with perfusable microchannels seamlessly underneath the gel matrices.

### Alignment of collagen and cellular central axis in microchannel-integrated anisotropically organized collagen 3D culture platform

**Fig. 3.**
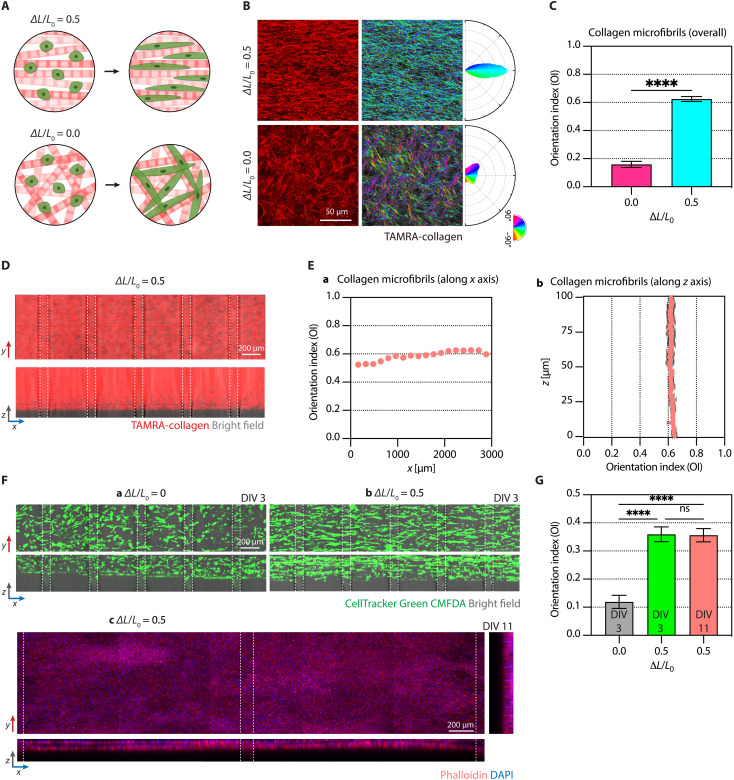
Alignment of collagen fibrils and cellular axes in the microchannel-integrated anisotropically organized collagen. (**A**) Schematic illustrations of anisotropically organized and randomly oriented collagen fibrils, followed by cellular alignment along the fibrils. (**B**) Confocal fluorescence images showing collagen fibrils (left), color-mapped images indicating orientation angles of the collagen fibrils (middle), and polar frequency histograms of the orientation angles (right). (**C**) Orientation indices (*29*) of uncompressed and precompressed collagen fibrils. Error bars indicate SD (*n* = 303; SD of 0.0215 and 0.0175 for uncompressed and precompressed, respectively). *n* denotes the number of images acquired from 101 *z* slices at three different regions. ****P < 0.0001. (**D**) Confocal fluorescence images of collagen fibrils over the entire area of the microchannel-integrated anisotropically organized 3D collagen (*xy* plane) and an orthogonal view merged with bright field (*xz* plane). White dashed lines indicate microchannel walls. (**E**) Orientation indices of collagen fibrils along the *x* (a) and *z* (b) axes analyzed from the images shown in (D). Error bars in (a) are buried within data (the number of analyzed images, *n* = 22 for each data point). Error bars in (b) indicate SD (the number of analyzed images, *n* = 3 for each data point). (**F**) Confocal fluorescence images showing human lung fibroblasts, stained with CellTracker Green CMFDA, in randomly oriented (DIV 3, a), collagen fibril–aligned (DIV 3, b; DIV 11, c) 3D matrices. The top and bottom panels indicate *xy*- and *xz*-plane views, respectively, with an additional *yz*-plane view in (c). White dashed lines indicate microchannel walls. DAPI, 4′,6-diamidino-2-phenylindole. (**G**) Orientation indices of the fibroblasts in uncompressed and precompressed matrices at DIV 3 and 11. Error bars indicate SD (the number of analyzed images, *n* = 4; SD of 0.0235, 0.0262, and 0.024 for uncompressed, and precompressed at DIV 3 and 11, respectively). Not significant (ns) *P* = 0.9787 and ****P < 0.0001.

We verified whether the reconfigurable microchannels might affect the alignment of collagen fibrils. To assess the collagen alignment under the utmost condition, we set the precompressive strain magnitude (∆*L*/*L*_0_) to be 0.5 (Fig. 3A). Collagen fibrils within the entire 3D scaffold integrated with five microchannels aligned well in parallel to the strain direction along the *x* axis (Fig. 3, B to Ea). In contrast, the nondeformed case (i.e., ∆*L*/*L*_0_ = 0) resulted in random distributions of the fibrils (Fig. 3, B and C). Moreover, the fibrils under the high strain magnitude also aligned well through the gel thickness along the *z* axis, representing up to 100 μm (Fig. 3, D and Eb). The orientation index, quantification of collagen fibrils’ orientation, revealed that *∆L*/*L*_0_ of 0.5 allowed for a consistently high degree of alignment with a mean index of 0.624 ± 0.018 (cf. 0.159 ± 0.021 for *∆L*/*L*_0_ = 0), regardless of lateral (*x*; Fig. 3Ea) and vertical (*z*; Fig. 3Eb) directions. These data indicate the robustness and uniformness of deformation-induced collagen alignment and microchannel integration.

The alignment of collagen fibrils ultimately served as 3D contact guidance for the sprouting and alignment of cells seeded in matrices. As a model cell, we embedded human primary lung fibroblasts in the fibril-aligned collagen with the underlying microchannels and observed cellular alignment. Similarly, the fibroblasts’ cytosolic bodies were initially round immediately after the strain release and elongated along the collagen fibrils (i.e., the *x* axis) at 3 days in vitro (DIV) with a slightly reduced mean orientation index of 0.359 ± 0.026 compared to collagen fibrils without seeding cells, while those in the nonaligned collagen showed a randomly oriented distribution with a mean index of 0.119 ± 0.024 (Fig. 3, F and G). In addition, we have consistently found that the alignment of human lung fibroblasts sustained for an extended period of up to DIV 11 without delamination or detachment of cell-seeded scaffolds (Fig. 3Fc). Specifically, the orientation indices of the fibroblasts at DIV 3 and 11 were almost identical (0.356 ± 0.024 for DIV 11; Fig. 3G) despite overpopulated fibroblasts due to proliferation. Similarly, our previous work (*40*) also showed that the alignment induction of muscle progenitor cells in muscle extracellular matrix (MEM) maintained the anisotropic structure and enhanced functional maturation of skeletal muscle over 10 days.

### Spatially resolved local delivery of molecules from microchannels to fibril-aligned collagen for selective treatments

**Fig. 4.**
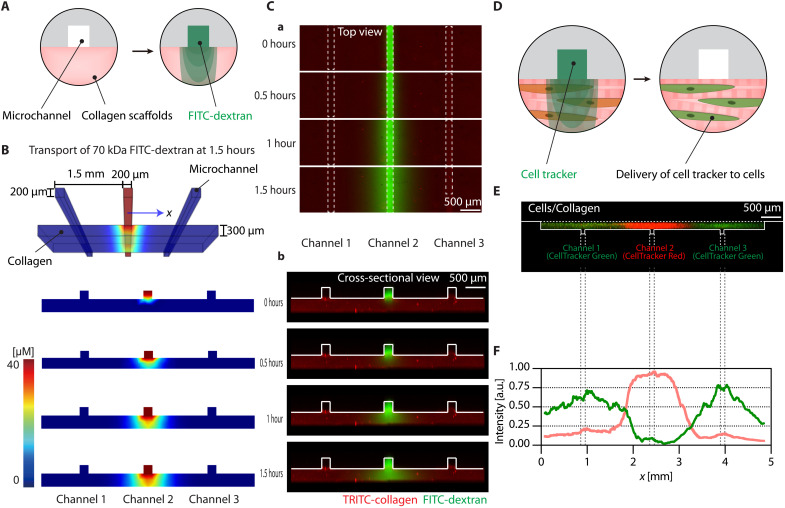
Spatially resolved local delivery of molecules from microchannels to fibril-aligned collagen for selective treatments. (**A**) A schematic illustration depicting local delivery through a microchannel to an anisotropically organized collagen 3D culture platform. (**B**) Computational simulation of local delivery of 40 μM of 70-kDa fluorescein isothiocyanate (FITC)–dextran into the collagen scaffold: 3D view at 1.5 hours and cross-sectional view at 0, 0.5, 1, and 1.5 hours. (**C**) Confocal fluorescence images displaying top (a) and cross-sectional (b) views for local delivery of 70-kDa FITC-dextran (green) via the center microchannel (channel 2) and diffusion into a tetramethyl rhodamine isothiocyanate (TRITC) (red)–labeled (*29*) collagen scaffold at 0, 0.5, 1, and 1.5 hours. White dashed lines in the top view and white solid lines in the cross-sectional view indicate microchannel walls and boundaries, respectively. (**D**) A schematic illustration depicting spatially resolved selective delivery of CellTracker to a collagen fibril–aligned scaffold seeded with cells. (**E**) A cross-sectional view of a confocal fluorescence image showing human lung fibroblasts labeled selectively in green near the side channels and red near the center channel after delivering CellTracker Green CMFDA via the side microchannels (channels 1 and 3) and CellTracker Red CMTPX via the center microchannel (channel 2). (**F**) Normalized *y*-mean intensity of the green- and red-labeled fibroblasts along the *x* axis from the image in (E). a.u., arbitrary units.

Microchannels integrated underneath the fibril-aligned collagen allow the spatially resolved, local delivery of various small and macromolecules into cells seeded within the 3D matrix. To confirm this capability, we first evaluated transient diffusion of 70-kDa fluorescein isothiocyanate (FITC)–dextran by convective delivery into a center microchannel of a three-channel–integrated chip and compared transport profiles with a computational calculation (Fig. 4, A and B). The computational simulation showed that the molecular diffusion occurred around the center microchannel in a spatially confined manner for 1.5 hours (Fig. 4B). Notably, the two side channels served as sinks (*32*) by delivering phosphate-buffered saline (PBS) or water that contained no solute of interest. In the experimentally identical setting, we observed a similar temporal evolution of localized diffusion into the fibril-aligned collagen scaffold (Fig. 4C).

For the following validation to confirm spatial control of the cellular microenvironment, we transported two different reagents into microchannels. Specifically, we delivered green CellTracker (465 Da) to the two side channels and red CellTracker (686 Da) to the center channel in direct contact with fibroblast-laden collagen (Fig. 4, D and E). Consequently, in 0.5 hours, the fibroblasts around the side channels were stained in green; in contrast, those around the center channel were stained in red (Fig. 4E). Cross-sectional profiles of fluorescence signals reaffirmed that we could stain cells in a region-specific manner (Fig. 4F). These results indicate that the integrated microchannels can selectively treat 3D cultured cells within monolithic fibril-aligned collagen.

### Alignment of neurites and in vitro chemical neuromodulation in microchannel-integrated anisotropically organized collagen 3D culture platform

**Fig. 5.**
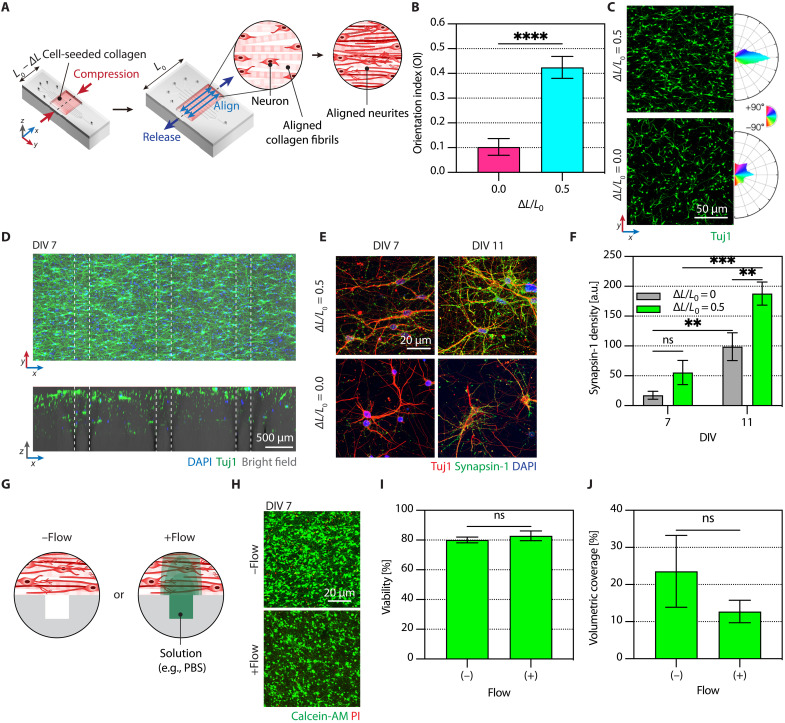
Aligned 3D neural networks in the microchannel-integrated anisotropically organized culture platform. (**A**) A schematic illustration depicting anisotropic neurite growth. (**B**) Orientation indices of the neurons cultured in uncompressed and precompressed collagen scaffolds. Error bars indicate SD (the number of analyzed images, *n* = 6; SD of 0.0336 and 0.0444 for uncompressed and precompressed, respectively). ****P < 0.0001. (**C**) Confocal fluorescence images showing the neurons (left) and polar frequency histograms of orientation angles (right). (**D**) Confocal fluorescence, merged with bright field, images of an aligned 3D neural network with rat primary cortical neurons at DIV 7: *xy* plane (top) and orthogonal *xz* plane (bottom). (**E**) Confocal fluorescence images of neuronal synapses (green), neurites (red), and nuclei (blue) in precompressed (top) and uncompressed (bottom) scaffolds at DIV 7 (left) and 11 (right). (**F**) Densities of synapsin-1 puncta from the images in (E). Error bars indicate SD. ns (between ∆*L*/*L*_0_ = 0 and 0.5 at DIV 7), ** (between ∆*L*/*L*_0_ = 0 and 0.5 at DIV 11), *** (between DIV 7 and 11 with ∆*L*/*L*_0_ = 0.5), and ** (between DIV 7 and 11 with ∆*L*/*L*_0_ = 0) *P* = 0.1260, 0.0088, 0.0002, and 0.0087, respectively. (**G**) A schematic illustration depicting the 3D culture of neurons without (left) or with (right) flow-through microchannels (i.e., fluidic shear stress to the cell-seeded scaffold). (**H**) Confocal fluorescence images of live (green) and dead (red) neurons under the flow conditions. Calcein-AM, calcein-acetoxymethyl; PI, propidium iodide. (**I** and **J**) Cell viabilities and volumetric coverage of the neurons under the conditions in (H). Error bars indicate SD [the number of analyzed images, *n* = 3; for −Flow, SD of 0.0194 (I) and 0.0969 (J); for +Flow, SD of 0.323 (I) and 0.03042 (J)]. ns *P* = 0.2672 (I) and 0.1378 (J).

We exploited our technology platform for reconstructing neural networks in vitro by embedding rat primary cortical neurons within microchannel-integrated, fibril-aligned collagen (Fig. 5A). The embedded neurons’ axons and dendrites sprouted following collagen fibrils along the precompression (*x*) axis already at DIV 3 (Fig. 5, B and C) and ultimately established 3D aligned neural networks at DIV 7 (Fig. 5D). Quantitatively, the mean orientation index calculated with the number of neuronal somata and neurites along the *x* axis resulted in 0.425 ± 0.044, revealing a notable alignment compared to those without the precompression (mean index of 0.103 ± 0.034) (Fig. 5, B and C). Furthermore, the alignment of collagen fibrils and subsequent neurite growth promoted synapse formation almost twofold at DIV 11 (Fig. 5, E and F).

Because convective flow-through microchannels induce molecular transport to fibril-aligned cellular collagen scaffolds, dominantly diffusion under a flow rate of 5 μl/min leading to high Pe and Bi numbers (*32*), the microfluidic biochemical modulations inevitably accompany fluidic shear stress (0.58 Pa) (*33*) to the cell-laden gels. Therefore, before chemical neuromodulation, we checked whether the existence of flow might alter the viability or growth of neurons. For this purpose, we delivered PBS and monitored neurons’ viability and volumetric coverage (Fig. 5G). We confirmed that the fluidic shear stress affected neither viability (Fig. 5, H and I) nor the volumetric coverage of neurons at DIV 7 (Fig. 5J).

**Fig. 6.**
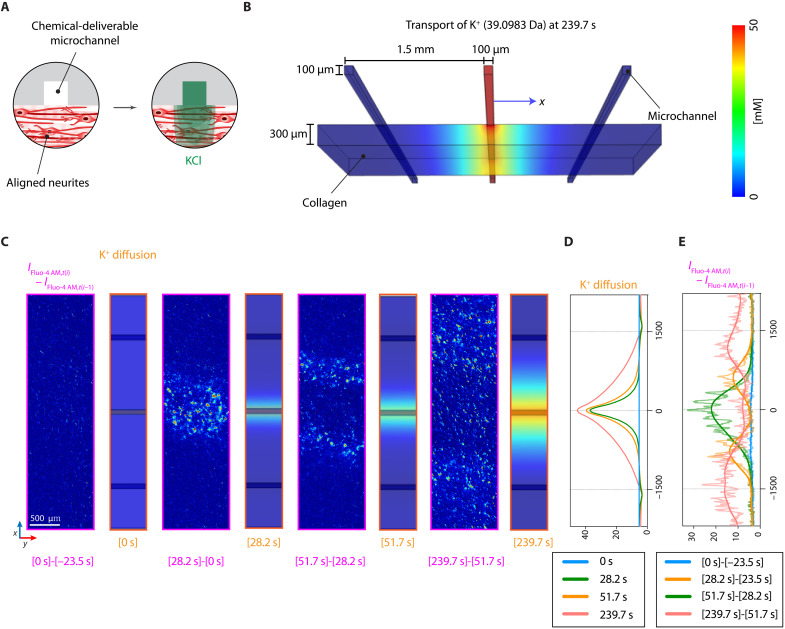
In vitro chemical neuromodulation of aligned 3D neural network. (**A**) Schematic illustration depicting microfluidic neuromodulation with KCl via the center channel to an aligned 3D neural network. (**B**) Computational simulation of 3D transient diffusion of K^+^ (39.0983 Da) into a collagen scaffold at 239.7 s. (**C**) Color-mapped differential confocal fluorescence images (left images in each panel) of Fluo-4 AM (Ca^2+^ signal) between two adjacent time frames [*I*_Fluo-4 AM,*t*(*i*)_ − *I*_Fluo-4 AM,*t*(*i*−1)_] and snapshots of the computational calculation (right images in each panel) from the solutions in (B) at 0 (*t*_0_), 28.2 (*t*_1_), 51.7 (*t*_2_), and 239.7 (*t*_3_) s. Scale bar, 500 μm. (**D**) *y*-Mean concentration of K^+^ along the *x* axis at *t*_i_. (**E**) [*I*_Fluo-4 AM,*t*(*i*)_ − *I*_Fluo-4 AM,*t*(*i*−1)_] along the *x* axis. Light and bold colors represent *y*-mean intensities and fitted curves generated by a smoothing option of LOWESS in Prism.

As a foundational capability of our microchannel-integrated, anisotropically organized 3D neuronal culture platform, we demonstrated spatially resolved chemical neuromodulation in neural networks in vitro (Fig. 6A and movie S1). For local stimulation of the neural network, we delivered 50 mM KCl dissolved in PBS through the center microchannel of a three-channel chip continuously over a neuromodulation time scale of ~240 s (Fig. 6B), which increased extracellular K^+^ concentration locally in the 3D neural tissue near the channel. The microfluidic delivery of KCl primarily induced immediate increases in neuronal depolarization of membrane potential through voltage-gated sodium (Na^+^) channels, Na_v_, and subsequent Ca^2+^ entry through voltage-gated Ca^2+^ channels (VGCCs). To visualize corresponding neural signals, we used a cell-permeable fluorescent Ca^2+^ indicator [i.e., Fluo-4 acetoxymethyl ester (AM)].

A computational calculation of K^+^ (39.0983-Da) diffusion in a collagen scaffold, assuming 100% ionization in PBS, predicted limited transport to ~600 μm from the center channel for ~240 s in reaching a threshold concentration of 20 mM to evoke observable increases in intracellular Ca^2+^ (Fig. 6, B to D) (*41*). However, the experimental result showed distinct temporal profiles of neurons’ Ca^2+^ signals (Fig. 6, C and E). More specifically, over an early time of 28.2 s after initiating the neuromodulation, the K^+^ diffusion profile was comparable to differential Ca^2+^ signals [i.e., *I*_Fluo-4 AM,*t*(*i*)_ − *I*_Fluo-4 AM,*t*(*i*−1)_]; we verified that these overlapping signals prolonged for up to 51.7 s (Fig. 6D). On the other hand, the differential fluorescent Ca^2+^ signals propagated from the center channel bidirectionally along the 3D aligned neural networks (movie S1) much rapidly further to >600 μm (positions of peak signals) at later time points (e.g., 51.7 s). Comparing the two peak intensities of the differential Ca^2+^ signal with tails of the K^+^ concentration at the same distance from the center channel at 239.7 s, we confirmed that the experimental peak signals originated evidently from the propagation along the aligned neural networks (Fig. 6, C to E) because ~8.2 mM K^+^ at *x* positions of ±1.2 mm is not enough to cause depolarization of Na_v_ (*42*–*45*). A summary by Rienecker *et al.* (*44*) listed various experimental conditions (i.e., duration and concentration) for the extracellular K^+^ treatment where 15 mM KCl was minimum for 0 to 5 min and lower 3 to 8 mM was for over 8 hours. Furthermore, on the basis of this propagation distance, we estimated the average speed of 3D Ca^2+^ signal propagation to be ~3.7 μm/s [cf. propagation of calcium signals by human umbilical vein endothelial cells cultured on a 2D substrate, ~17 μm/s (*46*); and muscle fiber conduction velocity by simulations, ~2 m/s (*47*)]. These results demonstrate that our local microfluidic stimulation could induce and analyze spatially resolved propagation of neural signals transmitting through synapses formed in the 3D neural networks in vitro.

**Fig. 7.**
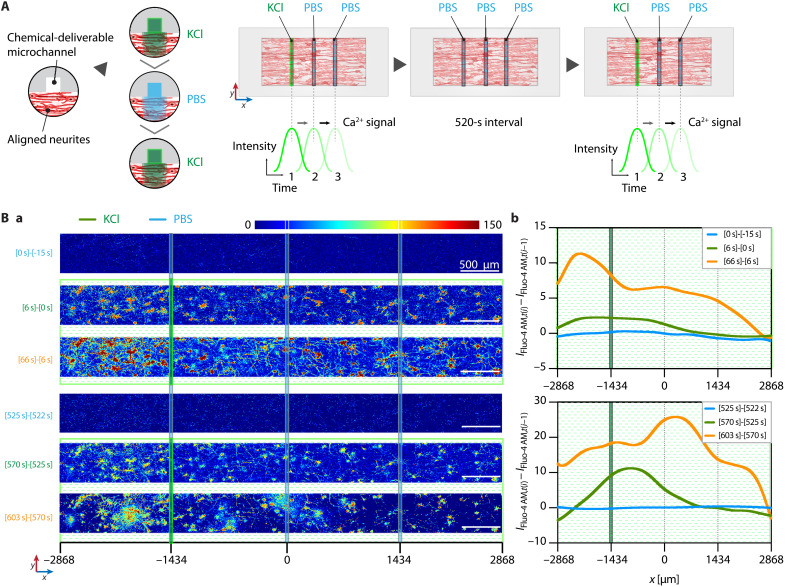
Spatiotemporally resolved chemical stimulation in aligned 3D neural network. (**A**) Schematic illustration depicting spatiotemporally resolved microfluidic neuromodulation by two consecutive pulsatile deliveries of KCl via the left microchannel to an aligned 3D neural network. (**B**) Color-mapped differential confocal fluorescence images of Fluo-4 AM (Ca^2+^ signal) between two adjacent representative time frames [*I*_Fluo-4 AM,*t*(*i*)_ − *I*_Fluo-4 AM,*t*(*i*-1)_] (a) and [*I*_Fluo-4 AM,*t*(*i*)_ − *I*_Fluo-4 AM,*t*(*i*−1)_] along the *x* axis after the first pulse: −18 (*t*_0_), 0 (*t*_1_), 6 (*t*_2_), and 66 (*t*_3_) s and the second pulse: 522 (*t*_0_), 525 (*t*_1_), 570 (*t*_2_), and 603 (*t*_3_) s (b). Scale bars, 500 μm. Curve profiles represent *y*-mean intensities generated by a smoothing option of LOWESS in Prism.

**Fig. 8.**
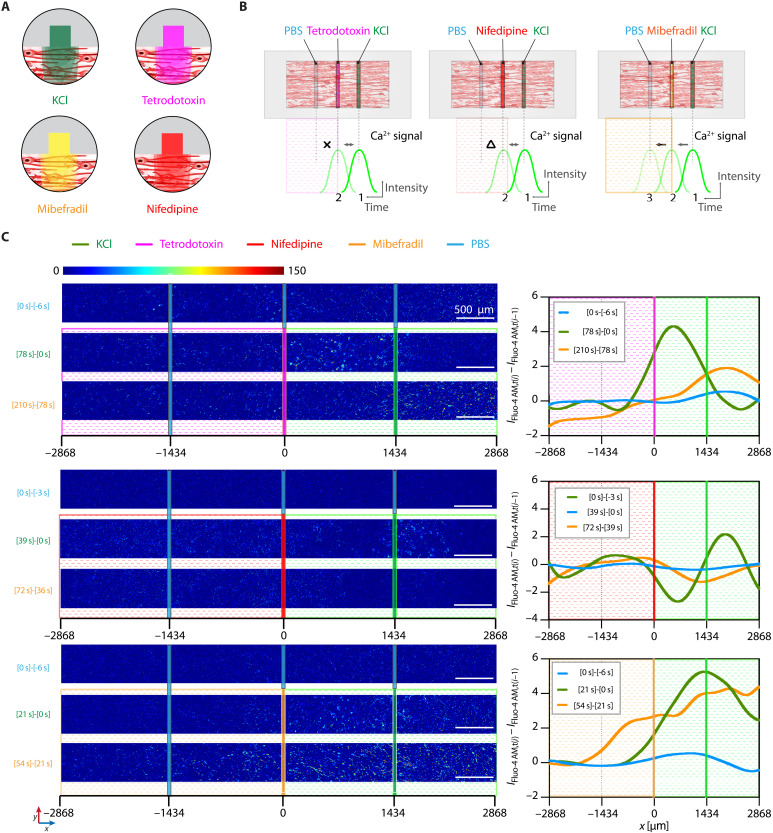
Spatially resolved chemical neuromodulation of aligned 3D neural network. (**A** and **B**) Schematic illustrations depicting spatially resolved microfluidic neuromodulation with KCl via the right microchannel and TTX, nifedipine, mibefradil via the center microchannel to an aligned 3D neural network. (**C**) Color-mapped differential confocal fluorescence images of Fluo-4 AM (Ca^2+^ signal) between two adjacent representative time frames [*I*_Fluo-4 AM,*t*(*i*)_ − *I*_Fluo-4 AM,*t*(*i*−1)_] (a) and [*I*_Fluo-4 AM,*t*(*i*)_ − *I*_Fluo-4 AM,*t*(*i*−1)_] along the *x* axis upon simultaneous treatments of TTX: −6 (*t*_0_), 0 (*t*_1_), 78 (*t*_2_), and 210 (*t*_3_) s; nifedipine: −3 (*t*_0_), 0 (*t*_1_), 39 (*t*_2_), and 72 (*t*_3_) s; and mibefradil: −6 (*t*_0_), 0 (*t*_1_), 21 (*t*_2_), and 54 (*t*_3_) s (b). Scale bars, 500 μm. Curve profiles represent *y*-mean intensities generated by a smoothing option of LOWESS in Prism.

Furthermore, we additionally demonstrated more functional features of our microchannel-mediated local neuromodulations in a spatiotemporally controlled manner: (i) temporally resolved, consecutive pulsatile deliveries of KCl through the left microchannel (Fig. 7 and movies S2 and S3) and (ii) spatially resolved inhibitions of Ca^2+^ signal propagation by delivering TTX, nifedipine, or mibefradil through the center channel during the simultaneous KCl stimulation through the right channel (Fig. 8 and movies S4 to S6).

The KCl treatment through the right microchannel induces depolarization of neurons adjacent to the KCl conduit, eliciting action potentials through voltage-gated Na^+^ channels. Subsequently, the action potential can propagate to neighboring neurons through either synaptic transmission or overflow of KCl from the conduit. The depolarization of the neurons would cause an opening of VGCCs, specifically high-voltage–activated (HVA) Ca^2+^ channels. Therefore, we could see the Ca^2+^ signal propagation following the KCl administration through a single conduit. To demonstrate the more sophisticated local neuromodulation in a more complicated experimental scheme, we tried to block the propagation of the Ca^2+^ signal to the left region of the anisotropically organized neural networks by administering a voltage-gated Na^+^ channel blocker (i.e., TTX) and two different VGCC blockers (i.e., nifedipine and mibefradil) through the center microchannel. Consistently with our expectation, we confirmed that both TTX and nifedipine, an HVA (L-type) Ca^2+^ blocker, substantially blocked the propagation of the Ca^2+^ signal (Fig. 8C and movies S4 and S5). On the contrary, mibefradil, a low-voltage–activated (T-type) Ca^2+^ channel blocker, did not effectively block the propagation (Fig. 8C and movie S6).

Generally, the number of synapses in a neural network would increase proportionally with neuron density in 2D cultures (*48*). Therefore, the seeding density of neurons in 3D collagen scaffolds will also affect the extent of the synapse formation. When testing two seeding densities (i.e., 4 × 10^6^ and 8 × 10^6^ cell/ml), we observed nearly no difference in the Ca^2+^ signal propagation. This result suggests that the seeding densities used to reconstruct the 3D aligned neural networks allowed for sufficient synapse formation and functional connectivity across the networks. Similarly, as reported in our previous work (*49*), where the seeding density was 1 × 10^7^ cell/ml, synaptic connections started to show from DIV 7 and much more active functional connective from DIV 10. These findings further validate the applicability of our microchannel-medicated, spatiotemporally resolved neuromodulation for inducing and blocking the propagation of neuronal activity within anisotropically organized networks.

## DISCUSSION

Now, no platform exists that allows for local delivery and selective treatment while anisotropically organizing 3D neural networks in vitro. Although recent developments of MPS related to 3D neural networks were compatible for downstream analyses with external tools, including 3D microelectrode arrays (MEAs) (*49*, *50*), 3D patch clamps (*51*), and electron microscopes (*31*), these devices do not feature any embedded microstructure permitting local delivery to a specific location of 3D neural networks in vitro. In contrast, we have integrated microchannels into the 3D neural network-laden collagen to achieve spatially resolved neuromodulation. A prominent advantage of our technology platform is that the microchannels can serve as sources and sinks of soluble factors and thus spatiotemporally control the microenvironment over an entire 3D neural network. For instance, the transport of biochemicals can be localized within regions of interest near microchannels in the network. Consequently, we can conceive studies investigating functional connectivity in vitro, such as region-to-region connections and long-range communications, by monitoring spatially resolved signals along aligned neural networks.

To integrate microfluidic channels simultaneously and seamlessly during the 3D alignment of collagen fibrils in a single device, we had to consider crucial design parameters (i.e., depth, *d*, and initial width, *w*_0_, of microchannels) controlling the wetting physics of sol-phase collagen on the open ridge structure in a micrometer scale during the precompressed state. More specifically, we temporarily reduced the “reentrant angle” (*52*) by deforming *w*_0_ to smaller *w*_d_, where sol-phase collagen’s contact angle and, thus, surface tension at the gel-PDMS interface exceeded the reentrant angle. In other words, we exploited the “capillary burst valve” principle to prevent the gel from filling the microchannels. Accordingly, we could classify the zones under conditions of the design parameters capable of patterning aligned collagen fibrils without blocking the microchannels by computational simulations followed by experimental verifications.

The aligned neural networks have become almost essential in fundamental studies of neurobiology and models of neurodegenerative diseases in vitro. For instance, our microchannel-integrated anisotropically organized collagen 3D culture platform can facilitate analyzing developing neural circuits, including synapse formation, over a long term (e.g., ~4 weeks) with spatiotemporal control of various biochemical factors in pluripotent stem cell (e.g., induced pluripotent stem cell and embryonic stem cell)–seeded matrices. Another readily feasible example is to selectively treat okadaic acid (OA) within a particular region of 3D neural networks in vitro; OA induces phosphorylated tau (pTau) in neurons (*53*–*55*). Because pTau spreads to neural networks via synaptic connections and causes tauopathy (*56*, *57*), this approach would allow for modeling tauopathies, a considerable class of neurodegenerative disorders (*58*) including Alzheimer’s disease (*59*), frontotemporal dementia with parkinsonism-17 (*60*), Pick’s disease (*61*), and progressive supranuclear palsy (*62*), in a more pathophysiologically relevant microenvironment. Moreover, because synucleinopathy has been attributed to synaptic transmission of α-synuclein (*63*, *64*), our platform could contribute to investigating the detailed mechanism regarding transsynaptic propagation of α-synuclein. Our platform could more versatilely extend applications to long-range communications between regions, such as the cortico-striatal pathway associated with various neurodegenerative diseases (e.g., Parkinson’s and Alzheimer’s).

To validate the capability of our platform in neuromodulation studies, we conducted additional spatiotemporally resolved neuromodulation by applying neurostimulant, KCl, and Ca^2+^ signal inhibitors through different microchannels. As shown in Figs. 7 and 8, the delivery of KCl through the right channel induced activation and propagation of Ca^2+^ signals, but such propagation was inhibited when we delivered TTX, nifedipine, and mibefradil through the center microchannel. These results imply that our platform enables delivering counteracting chemicals in a spatiotemporally resolved manner. Combined with multiple inlets for compartmentalizing distinct cell populations seeded in collagen (*29*), the multimicrochannel feature would enable independent, selective viral infection in various regions in the aligned neural tissue. Accordingly, this approach could permit elaboration on synaptic structural stability by spatiotemporally controlling the expression of synaptic proteins at presynaptic axon terminals and postsynaptic dendritic boutons. More specifically, spatially resolved transduction of mammalian green fluorescent protein (GFP) reconstitution across synaptic partners (mGRASP) (*65*, *66*) would lead to expressing functional complementation proteins in two distinct neuronal or interfacial regions. At synapses between pre–mGRASP-expressing and post–mGRASP-expressing neurons, two nonfluorescent split-GFP fragments tether to the synaptic cleft to visualize synapses. Therefore, our platform could serve as an unprecedented tool for the real-time analysis of synaptic structure and transmission in 3D neural networks in vitro.

More recently, genetically encoded fluorescent protein sensors have gained attention because of their capability to sensitively detect the activation of neurotransmitter receptors in the G protein–coupled receptor family (*67*–*69*). In a dopamine-receptor biosensor, for example, a circularly permuted fluorescent protein is inserted in the intracellular loop of the dopamine receptors, which can markedly increase its fluorescence when the receptor becomes activated upon dopamine binding. Thus, we can visualize specific neurotransmission, such as dopaminergic signals, which is a crucial element of signal transmission in functional neural networks (*70*). We are now applying these genetically encoded biosensors to our 3D neural networks in vitro to investigate further the complex mechanism of neurotransmission upon different levels or combinations of neurotransmitters.

In addition, using other cell types is readily feasible; for instance, our microchannel-mediated delivery of chemicals can display muscle contraction in response to local stimuli. Another example would be the construction of neurovascular units by exploiting the microchannels as brain endothelial templates; this model could serve as an in vitro tool for preclinical evaluations of drugs and drug candidates.

While our alignment technique readily applies to other matrix conditions [e.g., decellularized ECMs (dECMs)], an essential requirement is that fibrous matrix components such as collagen type I should be sufficiently abundant in a matrix. For example, we have previously applied this principle to the MEM (*40*). Another example is to create a composite matrix by adding collagen type I in the brain dECM, where collagen type I is not a dominant component (*38*). A potentially more impactful example would be to use a cocktail of human brain dECM components (*19*) and add collagen type I if needed.

Despite the advancements reported in this study, we have identified the following limitations of our technology platform. First, the alignment technique by predeformation and releasing a gel-containing PDMS substrate applies only to fibrous hydrogels. Collagen type I, one of the biologically natural gels, has been the best candidate primarily because of its fibrillogenesis kinetics exhibiting a readily operational (relatively long) window for nucleation (0 to 3 min), formation of short microfibrils (4 to 15 min), and fibril growth (15 to 60 min). Alternatively, introducing synthetic hydrogels (*71*, *72*) and tweaking their gelation or crosslinking kinetics could overcome the issue of material versatility. Second, our methodology has offered bidirectional connectivity in 3D neural networks in vitro; in contrast, most neural circuits in vivo (e.g., the hippocampal circuit from dentate gyrus to CA1 via CA3) are known to be unidirectional. However, this functional directionality between regions is not limited only to our technology platform, and no other work has reported reconstructing 3D unidirectional connectivity in vitro. Next, direct measurements of neurons’ electrophysiological signals, occurring on the order of 10^0^ to 10^1^ ms, are difficult solely with our platform. Instead, more detailed studies, including elucidating neural circuit dynamics in vitro, have become available with an external tool such as the 3D MEA (*49*). Last, limitations of our technology platform for stimulating organized neural networks would include spatial and temporal resolutions of neuromodulation. The spatial resolution over substantially shorter length scales (e.g., almost down to a single somatic body of approximately 10 to 20 μm) would be challenging due to a limit originated from the fabrication of a denser array of microchannels with *w*_0_ and *d* of 10 to 20 μm. In addition, fluidic interfaces and their operations with the temporal resolution of a few seconds would be technically tricky unless we build an automated multichannel microfluidic dispenser.

We have developed a microchannel-integrated anisotropically organized 3D collagen culture platform. We confirmed the fabrication principles and design parameters using computational simulations and relevant experiments. The microfluidic channels seamlessly integrated underneath the 3D fibril-aligned neural networks allowed the localized delivery of biochemicals. The spatially resolved chemical neuromodulation with KCl enabled the visualization of Ca^2+^ signal propagation over time, and we could estimate propagation speed along aligned 3D neural networks. We anticipate that our technology will be a powerful platform to elucidate the roles of structural and functional neural connectivity and the progression of neurological diseases.

## MATERIALS AND METHODS

### Experimental design

This study aimed to develop an aligned neural network model integrated with perfusable microchannels. Specifically, we exploited the precompression mode (*29*) to reconstruct an anisotropically organized 3D collagen matrix. At the same time, we implemented the perfusable microchannels seamlessly underneath the fibril-aligned collagen to enable the localized delivery of molecules into specific regions of neural tissue, thus allowing the spatially controlled chemical neuromodulation in vitro. Our fabrication approach aimed to control the closing and reopening of the microchannels to prevent undesired permeation of sol-state collagen by using the elastic properties of PDMS. To determine optimal design parameters of depth (*d*) and initial width (*w*_0_) of microchannels, we developed a computational model simulating anisometric changes of microchannels’ cross-sectional structure under compression and verified representative conditions experimentally. Then, we engineered in vitro chemical neuromodulation of the aligned 3D neural network by demonstrating the capability of spatially resolved local delivery and selective treatment to stimulate the 3D neural networks and observe temporal evolutions of neuronal Ca^2+^ signal propagation.

### Fabrication of reconfigurable microchannel-integrated PDMS chip

To fabricate the reconfigurable microchannel-integrated PDMS chip, we first produced two masters for the PDMS replica: a duralumin master for the top layer with a rectangular through-hole referred to as a PDMS stencil and an SU-8 master for an intermediate bottom layer patterned with three or five microchannels merging into one (Fig. 1D1). We micromilled a duralumin block to create a rectangular well (5 mm by 8 mm) with a depth of 400 μm for the PDMS stencil. For the bottom PDMS layer, we spin-coated SU-8 2150 (MicroChem) on a silicon wafer to reach a target thickness of 100, 200, or 250 μm and exposed ultraviolet (365 nm) through a photomask according to the MicroChem’s standard protocol.

We coated trichloro (1H,1H,2H,2H-perfluorooctyl) silane (Sigma-Aldrich) on both masters before pouring a degassed mixture of PDMS (SYLGARD 184; Dow Corning) and the curing agent at a weight ratio of 10:1. We placed a glass slide slowly onto the PDMS-poured duralumin master and put weight to eliminate generating an interfacial layer. This step should proceed cautiously to avoid air bubbles at the glass-PDMS interface. We peeled off PDMS replicas after curing the PDMS mixture at 80°C for 2 hours. The thicknesses of the PDMS stencil and intermediate bottom layer were 400 μm and 5 mm, respectively. We trimmed the films off with forceps (sharp tweezers) and a scalpel if any residual PDMS films formed on the through-hole. We note that it is essential to prepare a thick, safely, at least ~10 mm, PDMS chip to prevent a PDMS chip from undesirably bending or wrinkling during the precompression. Therefore, we produced a 5-mm-thick PDMS block as a base layer on a petri dish. Another reason for separately preparing this base layer is to avoid failure as much as possible when peeling off the microchannel-patterned layer; for example, peeling off a 10-mm-thick bottom layer at once occasionally causes torn areas on the microchannel pattern. We immediately bonded the base layer on the flat side of the intermediate bottom layer after treating both layers with O_2_ plasma at radiofrequency power of 8 W for 40 s (Femto Science). Then, we bonded the PDMS stencil onto the 10-mm-thick bottom layer’s microchannel-patterned side while aligning the centers of the microchannels and the through-hole.

For microfluidic delivery of chemicals, we flipped the entire PDMS chip with fibril-aligned collagen upside down and placed it on a glass slide. Combined with withdrawal from the outlet at a flow rate of 5 μl/min, this approach allowed for a liquid-tight interface. Therefore, we created through-holes for the in- and outlets. For the chemical neuromodulation (Fig. 6), we punched all the inlet and outlet holes with a 0.4-mm biopsy punch and connected Tygon tubing (S-54-HL; Saint-Gobain Performance Plastics; inner and outer diameters of 1.016 and 1.778 mm, respectively). For other experiments (Figs. 4 and 5), we punched the inlet holes with a 5-mm biopsy punch, serving as chemical-loadable reservoirs.

### Polydopamine coating for adhesion of collagen on PDMS surfaces

For collagen adhesion on PDMS, we coated the inner surfaces of the PDMS well (5 mm by 8 mm by 0.4 mm) with polydopamine by applying dopamine hydrochloride (2 mg/ml; Sigma-Aldrich) dissolved in 10 mM tris-HCl buffer (pH 8.5) (Fig. 1D2). Achieving adhesion between collagen and the surfaces of the PDMS well is critical, not only for sealing the perfusable microchannels but also for preventing collagen delamination. The polydopamine coating followed by sol-phase collagen allowed for the creation of covalent bonds between the polydopamine-coated PDMS and collagen (*29*, *38*, *40*, *73*, *74*).

On the basis of our practical experience for years, the reproducibility of successful fabrication of collagen fibril–aligned scaffolds with PDMS chips has been more than 90%. However, we have unexpectedly found that choosing the timing of sol-phase collagen onto the polydopamine-coated PDMS well affects the fabrication reproducibility substantially. We recommend the following steps: (i) coat polydopamine for 3 hours at room temperature, (ii) aspirate residual polydopamine and rinse the well three times with deionized water, (iii) air-dry within a clean bench overnight, and (iv) load the sol-phase collagen. An example leading to fabrication failure occurs when loading the sol-phase collagen immediately after the polydopamine coating (step ii). Some complicated factors associated with wettability around interfacial edges of the PDMS well could cause undesired wicking and substantial loss of collagen in the central region after the release.

### Fabrication of fibril-aligned 3D collagen integrated with microchannels

To fabricate fibril-aligned 3D collagen integrated with microchannels, we proceeded with the following steps:

(Caution) Use the PDMS chips coated with polydopamine and air-dried overnight to maximize the reproducibility for the fabrication of fibril-aligned 3D collagen integrated with microchannels.

1) Compress the PDMS chip along the *x* axis from *L*_0_ to *L*_0_ − ∆*L* with a precise vise (PMV-3 N; Giant; 73 mm by 35 mm by 210 mm), closing the microchannels (Fig. 1D3).

2) Load either cell-seeded (Figs. 3 to 8) or cell-free (Figs. 2 to 4) collagen into the predeformed, polydopamine-coated well (Fig. 1D4). See descriptions in the next section for the preparation of collagen solutions.

3) Allow partial fibrillogenesis for 5 min at room temperature (Fig. 1D4).

4) Release strain by restoring the precompressed PDMS chip to its original configuration from *L* − ∆*L* to *L* along the *x* axis, aligning short collagen microfibrils, and reopening the microchannels (Fig. 1D5).

(Note) With ∆*L*/*L*_0_ of 0.5, strain applied to the collagen scaffold during this release step (∆*L*_collgen_/*L*_0,collagen_) is −0.3.

5) Complete fibrillogenesis in a humidified incubator (37°C and 5% CO_2_) for 30 min (Fig. 1D6).

6) Apply 5 ml of medium for 3D cell culture.

### Computational simulations by FEM

We performed computational calculations by finite element method (FEM) (COMSOL Multiphysics, COMSOL) for the following analyses: (i) the structural deformation of open ridges of PDMS microchannels under compression (Fig. 2, B to D), (ii) the spontaneous permeation of collagen into the deformed microchannels (Fig. 2, F and H), and (iii) molecular transport to collagen via microchannels (Figs. 4B and 6B).

We used the 2D static solid mechanics module for the deformation analysis. We set Young’s modulus and Poisson’s ratio of PDMS to be 750 kPa and 0.49, respectively. Then, we applied prescribed displacement from 0 to 0.5 to an elastic domain (i.e., PDMS). To simulate the permeation of collagen, we redrew computational solutions acquired from the deformation analysis to triangular or trapezoidal shapes. Then, we used the 2D two-phase transport module to predict the spontaneous permeation of sol-phase collagen into deformed microchannels filled with air by varying the two structural parameters: depth (*d*) and initial width (*w*_0_) of a microchannel. Preset constants were (100 μm by 100 μm) for Fig. 6 and (200 μm by 200 μm) for Fig. 4.

We used the transport of the diluted species module to model transient 3D diffusion of FITC-dextran (70 kDa) and K^+^ (39.0983 Da) in a porous matrix via a permeable boundary between a microchannel and the matrix. We calculated diffusion coefficients of the two aqueous species in a microchannel, *D*_c_, with the following equation

$$D_{c}=1.013\times 10^{-8}{\left(MW\right)}^{-0.46}\;[m^{2}/s]$$

Accordingly, *D*_c,FITC−dextran_ and *D*_c,K^+^_ were 5.98 × 10^−11^ and 1.88 × 10^−9^ m^2^/s, respectively. Assuming that the porous matrix had porosity of 0.8, we set diffusivity in gel, *D*_g_, to be 0.8 *D*_c_. We applied a convective flow rate of 8.33 × 10^−11^ m^3^/s (5 μl/min) to aqueous solutions through the center channel and water through all the side channels. The concentrations of the two solutes delivered through the center channel were 40 μM for FITC-dextran and 50 mM for K^+^. These preset concentrations were identical to the experimental values used for Figs. 4B and 6, B to D.

### Rheological analysis

To investigate the rheological properties (i.e., storage and loss moduli) of collagen type I (2.5 mg/ml) and thus elucidate fibrillogenesis kinetics (Fig. 2I), we loaded cell-free, sol-phase collagen on a test bed of the rheometer (MSC 102; Anton Paar). We set the initial temperature of the test bed at 4°C for 3 min. Then, we increased the temperature to 37°C to initiate fibrillogenesis and measured moduli.

### Fluorescent labeling of collagen fibrils

To visualize collagen fibrils (Figs. 1A, 2H, 3, B and D, and 4C), we followed a previous protocol (*29*). Briefly, we incubated collagen scaffolds with 5 μM 5-carboxytetramethylrhodamine (Thermo Fisher Scientific) in PBS for 1 hour at room temperature and rinsed three times with PBS before confocal laser scanning microscopy (LSM700; Zeiss).

### Culture and labeling of fibroblasts

We used human lung fibroblasts (Lonza, catalog no. CC-2512) cultured in fibroblast growth medium (Lonza) in a humidified incubator at 37°C and 5% CO_2_. For the experiments (Fig. 3F), we used the fibroblasts from the 10th passage. We expanded the cells to subcultures when reaching 80% confluence before use.

To analyze the extent of alignment (Fig. 3G) and to demonstrate the spatially resolved control of the tissue microenvironment (Fig. 4, D to F), we labeled the fibroblasts with CellTracker Green (CMFDA; Thermo Fisher Scientific) and CellTracker Red (CMTPX; Thermo Fisher Scientific). We delivered 1 μM of CellTracker Red through the center channel and CellTracker Green through the two side channels for 30 min, followed by *z*-stack imaging of the confocal microscopy with a 1-μm interval up to 100 μm.

To stain F-actin of fibroblasts at DIV 11, we first fixed 3D engineered constructs in 4% (w/v) paraformaldehyde (PFA). Next, we proceeded by blocking with 2% (w/v) bovine serum albumin (BSA) and permeabilization with 0.1% (v/v) Triton X-100 in PBS. We performed these three steps at room temperature. Then, we incubated the 3D engineered tissues sequentially with Alexa Fluor 594 Phalloidin (1:1000; Thermo Fisher Scientific, catalog no. A12381) diluted in the blocking solution at room temperature overnight. We stained nuclei with Hoechst 33342 (1:5000; Invitrogen; H3570). We rinsed all samples with PBS between the incubation steps.

### Isolation and culture of primary cortical neurons

We purchased pregnant Sprague-Dawley rats (embryonic day 18; SAMTAKO) and euthanized perinatal embryos for isolating primary cortical neurons, following a previous protocol (*29*). We conducted all procedures according to the animal welfare guidelines approved by the Institutional Animal Care and Use Committee of the Korea Institute of Science and Technology. Briefly, we dissected the entire cortex as an intact structure from the embryos’ brains under a dissection microscope. Next, we treated the cortex with papain (Miltenyi Biotec) and serially triturated. Then, we seeded dissociated cells, predominantly neurons, within collagen; see the following section for more details. We cultured the neurons in medium consisting of neurobasal medium supplemented with 2% (v/v) B27 supplement (Invitrogen), 2 mM GlutaMAX I (Thermo Fisher Scientific), and 1% (v/v) penicillin-streptomycin (Thermo Fisher Scientific) in a humidified incubator at 37°C and 5% CO_2_. During the 3D culture, we replaced half the medium with fresh medium every 2 to 3 days.

### Preparation of cell-free or cell-seeded collagen

We prepared either cell-free or cell-seeded collagen at a final concentration of 2.5 mg/ml with the commercially available collagen type I (Corning Collagen I, High Concentration, Rat Tail; Corning, product number 354249). Depending on the concentration of collagen stock solution (8 to 11 mg/ml; variable by lots), we first transferred either 110 to 160 mg or 110 to 160 μl of the stock collagen to a 1.5-ml microtube. Then, we added 50 μl of 10× Dulbecco’s modified Eagle’s medium (DMEM; Sigma-Aldrich) and 1× DMEM to match a total volume of 445 μl, followed by gently mixing. To adjust pH to ~7, we added 5 μl of 0.5 M NaOH. The mixture color changed from yellow to pink by phenol red in the mixture. Last, we added either 50 μl of cell-suspended DMEM for cell-seeded collagen or 1× DMEM for cell-free collagen to reach the seeding density of 4 × 10^6^ cell/ml, followed by gently mixing. Immediately, we loaded the cell-seeded or cell-free collagen into the PDMS well while keeping the neutralized collagen on ice to minimize undesired gelation.

### Cell viability assay

We assessed the cell viability by incubating cell-seeded collagen scaffolds in PBS containing calcein-acetoxymethyl (0.5 mg/ml; Invitrogen) and propidium iodide (2 mg/ml; Sigma-Aldrich) for 30 min in a humidified incubator at 37°C and 5% CO_2_. After washing with PBS, we performed confocal laser scanning microscopy to acquire green and red fluorescence images. We used these images to count live (green) and dead (red) cells (Fig. 5, H and I) and to estimate volumetric coverage (Fig. 5J).

### Immunofluorescence staining

To stain neurites and synaptic proteins (Fig. 5, C to E), we first fixed 3D engineered neural constructs in 4% (w/v) PFA. Next, we proceeded by blocking with 2% (w/v) BSA and permeabilization with 0.1% (v/v) Triton X-100 in PBS. We performed these three steps at room temperature. Then, we incubated the 3D engineered tissues sequentially with primary and secondary antibodies diluted in the blocking solution at 4°C overnight. We used the following primary antibodies: mouse anti–β-tubulin III (TuJ1) (1:1000; Sigma-Aldrich, catalog no. T8578, RRID: AB_1841228), chicken anti–microtubule-associated protein 2 (1:500; Abcam, catalog no. ab5392, RRID: AB_2138153), rabbit anti–synapsin-I (1:2000; Cell Signaling Technology, catalog no. 5297, RRID: AB_2616578), and mouse anti–postsynaptic density protein (PSD)-95 (1:1000; Thermo Fisher Scientific, catalog no. MA1-045, RRID: AB_325399). We used the following secondary antibodies: goat anti-mouse immunoglobulin G (IgG) (H+L), Alexa Fluor 488 (Thermo Fisher Scientific, catalog no. A-11001, RRID: AB_2534069); goat anti-rabbit IgG (H+L), Alexa Fluor 594 (Thermo Fisher Scientific, catalog no. A-11012, RRID: AB_2534079); goat anti-chicken IgY (H+L), Alexa Fluor 594 (Thermo Fisher Scientific, catalog no. A-11042, RRID: AB_2534099); goat anti-guinea pig IgG (H+L), Alexa Fluor 594 (Thermo Fisher Scientific, catalog no. A-11076, RRID: AB_2534120); goat anti-mouse IgG (H+L), Alexa Fluor 647 (Thermo Fisher Scientific, catalog no. A-21236, RRID: AB_2535805); and goat anti-rat IgG (H+L), Alexa Fluor 647 (Thermo Fisher Scientific, catalog no. A-21247, RRID:AB_141778). We stained nuclei with Hoechst 33342 (1:5000; Invitrogen, H3570). We rinsed all samples with PBS between the incubation steps.

### Spatiotemporally resolved local neuromodulation in vitro and post-image analysis

After reconstructing an aligned 3D neural network by culturing rat primary cortical neurons within a collagen fibril–aligned scaffold integrated with three microfluidic channels for 14 days, we applied 1 μM of a cell-permeable Ca2^+^ indicator, Fluo-4 AM (Thermo Fisher Scientific) diluted in the culture medium and incubated for 30 min. Next, we placed the PDMS chip on a confocal microscope (LSM700; Zeiss) and loaded 50 mM KCl dissolved in the culture medium and PBS into two external reservoirs connected with the Tygon tubing. Then, after initiating the acquisition of time-series images [red-green-blue (RGB); 2048 × 512 pixels] with an interval of 4.7 s, we delivered 50 mM KCl to a central region of the 3D neural network via the center channel by withdrawing from the outlet at a flow rate of 5 μl/min. We acquired the time-series images for up to 465.3 s.

For the temporally (Fig. 7) and spatially (Fig. 8) resolved neuromodulation, we placed the PDMS chip on a confocal microscope (LSM800; Zeiss), loaded PBS into three external reservoirs connected with the Tygon tubing, and withdrew from the outlet at a flow rate of 5 μl/min. When initiating the temporal modulation, we loaded 20 μl of 50 mM KCl dissolved in culture medium to one of the three external reservoirs connected to the left microchannel and allowed for gravity-driven delivery (i.e., an instantaneous shot). The rest two reservoirs remained filled with PBS. With an intermediate loading of PBS for 520 s as an interval, we applied another instantaneous shot of KCl to the identical microchannel. For the spatial modulation, we replaced PBS preloaded in the middle reservoir with 20 μl of 500 nM TTX, 100 μM nifedipine, and 10 μM mibefradil, respectively, while administrating the right reservoir with 20 μl of 50 mM KCl dissolved in culture medium. We acquired time-series and tile scan images (RGB; 5735 × 512 pixels) with an interval of 3 s until the end of each batch.

To obtain profiles of differential Ca^2+^ signals, we first selected a subset of time frames showing noticeable changes in the Fluo-4 AM signal before and after the local delivery of KCl; the selected time frames were −23.5, 0 (*t*_0_), 28.2 (*t*_1_), 51.7 (*t*_2_), and 239.7 (*t*_3_) s. For the temporal modulation, representative time frames were −15, 0 (*t*_0_), 6 (*t*_1_), 66 (*t*_2_), 522 (*t*_3_), 525 (*t*_4_), 570 (*t*_5_), and 603 (*t*_6_) s. Representative time frames for the TTX, nifedipine, and mibefradil treatments were [−6, 0 (*t*_0_), 78 (*t*_1_), and 210 (*t*_2_)], [−3, 0 (*t*_0_), 39 (*t*_1_), and 72 (*t*_2_)], and [−6, 0 (*t*_0_), 21 (*t*_1_), and 54 (*t*_2_)], respectively. Next, using ImageJ, we extracted the green channel (8-bit monochrome) and cropped the images to align the center with the center channel (1800 × 512 pixels). Then, we ran a custom MATLAB (MathWorks) code to subtract an image at a previous adjacent time frame from one at a time frame of interest [*I*_Fluo-4 AM,*t*(*i*)_ − *I*_Fluo-4 AM,*t*(*i*−1)_] and color map with an image scale from 0 to 150 (code S1). We referred to the resultant images as differential Ca^2+^ signals (Fig. 6C). In addition, we averaged the fluorescence intensity of each resultant image along the *y* axis (512 pixels) to create the profiles of *y* mean [*I*_Fluo-4 AM,*t*(*i*)_ − *I*_Fluo-4 AM,*t*(*i*−1)_] along the *x* axis (1800 pixels; Figs. 6E and 8C; code S2). We generated fitted curves using a smoothing option of LOWESS in Prism (GraphPad).

### Statistical analysis

We determined the statistical significance from a minimum of three independent experiments and performed all statistical analyses in Prism (GraphPad Software). After confirming that data passed normality and variance tests, we determined statistical significance using the ANOVA to compare means of more than two samples or groups and the *t* test to compare means between two samples. To represent the significance of the comparisons, **, ***, and **** denote *P* < 0.01, 0.001, and 0.0001, respectively. We also specified individual *P* values in the figure captions.

## Supplementary Materials

### This PDF file includes:

Fig. S1 Legends for movies S1 to S6 Codes S1 and S2

## Notes

View/request a protocol for this paper from *Bio-protocol*.
