# Supplementary material for: Integration of reconfigurable microchannels into aligned three-dimensional neural networks for spatially controllable neuromodulation
Source: Sci Adv. 2023 Mar 10;9(10):eadf0925. doi: 10.1126/sciadv.adf0925 (PMC10005277; doi:10.1126/sciadv.adf0925)
Supplement: Supplementary file 1 — Fig. S1 Legends for movies S1 to S6 Codes S1 and S2 [file sciadv.adf0925_sm.pdf]

Science Advances
AAAS

Supplementary Materials for

Integration of reconfigurable microchannels into aligned three-dimensional neural networks for spatially controllable neuromodulation

Sohyeon Jeong *et al.*

Corresponding author: Nakwon Choi, nakwon.choi@kist.re.kr; Hong Nam Kim, hongnam.kim@kist.re.kr; Seokyoung Bang, seokyoung.bang@dongguk.edu; Seok Chung, sidchung@korea.ac.kr

Sci. Adv. **9**, eadf0925 (2023)
DOI: 10.1126/sciadv.adf0925

The PDF file includes:

Fig. S1
Legends for movies S1 to S6
Codes S1 and S2

Other Supplementary Material for this manuscript includes the following:

Movies S1 to S6

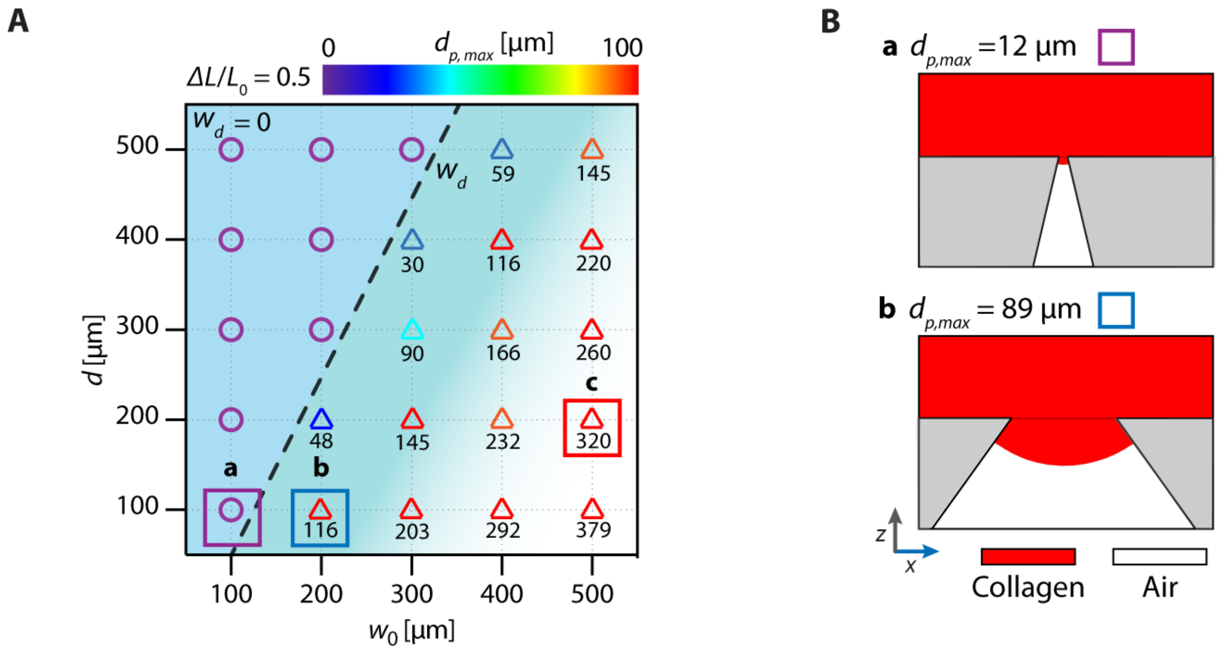

Fig. S1.

Design principles for integrating reconfigurable microchannels into collagen fibril-aligned 3D matrix. (**A**) A zone-marked contour plot displaying computational predictions for permeation of sol-phase collagen at 5 min into deformed vacant microchannels (w_d) under pre-compression (ΔL / L_0 = 0.5) with varying w_0 and d: $d_p = 0$ (◯) and $0 < d_p < d$ (△). (**B**) Images showing computational predictions of microchannel cross-sections. Collagen permeation and air are labeled red and white, respectively.

Movie S1.

Propagation of neuronal Ca^{2+} signals along the 3D aligned neural networks bidirectionally from the center microchannel delivered with KCl.

Movie S2.

Propagation of neuronal Ca^{2+} signals along the 3D aligned neural networks from the left microchannel delivered with the first pulse of KCl.

Movie S3.

Propagation of neuronal Ca^{2+} signals along the 3D aligned neural networks from the left microchannel delivered with the second pulse of KCl.

Movie S4.

Propagation and inhibition of neuronal Ca^{2+} signals along the 3D aligned neural networks from the right microchannel delivered with KCl, and the middle microchannel with TTX.

Movie S5.

Propagation and inhibition of neuronal Ca^{2+} signals along the 3D aligned neural networks from the right microchannel delivered with KCl, and the middle microchannel with Nifedipine.

Movie S6.

Propagation and inhibition of neuronal Ca^{2+} signals along the 3D aligned neural networks from the right microchannel delivered with KCl, and the middle microchannel with mibefradil.

Code S1.

Matlab code to create colormapped images in a frame of interest ($I_{\text{Fluo-4 AM},t(\text{i})}$ - $I_{\text{Fluo-4 AM},t(\text{i-1})}$)

```
clc; %Clear Command Window
clear all; %Remove items from workspace, freeing up system memory
close all;

%Open standard dialog box for selecting directory
dname_fl = uigetdir('any desired location on a computer to find images');

%List all tif files
cd(dname_fl);
img_path_fl=strcat(dname_fl,'/*.tif');
file_fl = dir(img_path_fl);
file_num_fl = length(file_fl);
file_name_fl = {file_fl.name};
file_date_fl = {file_fl.date};

file_num_fl

clims_fl=[0 150];

img_w=5735;
img_h=512;

 for i = 2:file_num_fl
    figure()
    img_fl=imread(file_name_fl{i})-imread(file_name_fl{i-1});
    imagesc(img_fl, clims_fl)
    colormap(jet)
    axis equal
    axis off
 end
```

Code S2.

Matlab code to analyze averaged fluorescence intensity from images and to create profiles of [$I_{\text{Fluo-4 AM},t(i)}$ - $I_{\text{Fluo-4 AM},t(i-1)}$] along the x-axis.

```
clc; %Clear Command Window
clear all; %Remove items from workspace, freeing up system memory
close all;

%Open standard dialog box for selecting directory
dname = uigetdir('any desired location on a computer to find images');

%List all tif files
cd(dname);
img_path=strcat(dname,'/*.tif');
file = dir(img_path);
file_num = length(file);
file_name = {file.name};
file_date = {file.date};

file_num

img_size=[];
t_ID={}
for i = 1:file_num
   img=imread(file_name{i});
   img_size=[img_size; size(img, 1) size(img, 2)]
   t_ID(i)=extractBetween(file_name{i}, "_t", ".")
end

img_sig=[];

x=1:img_size(i,2);
x2=(x-1)';

for i = 1:file_num
   img=imread(file_name{i});
   mean_img=mean(img(:,:,2));
   mean_img=mean_img';
   img_sig=[img_sig mean_img];
end

%Export to excel
T=table(x2, img_sig);
filename = 'mean_intensity.xlsx';
writetable(T,filename,'Sheet',1,'Range','A1')
```
